# Protein Kinase Signaling Networks Driven by Oncogenic Gq/11 in Uveal Melanoma Identified by Phosphoproteomic and Bioinformatic Analyses

**DOI:** 10.1016/j.mcpro.2023.100649

**Published:** 2023-09-19

**Authors:** Michael D. Onken, Petra Erdmann-Gilmore, Qiang Zhang, Kisan Thapa, Emily King, Kevin M. Kaltenbronn, Sarah E. Noda, Carol M. Makepeace, Dennis Goldfarb, Özgün Babur, R. Reid Townsend, Kendall J. Blumer

**Affiliations:** 1 Department of Biochemistry and Molecular Biophysics, Washington University in St Louis, St Louis, Missouri, USA; 2 Department of Medicine, Washington University in St Louis, St Louis, Missouri, USA; 3 Department of Computer Science, University of Massachusetts Boston, Boston, Massachusetts, USA; 4 Department of Cell Biology and Physiology, Washington University in St Louis, St Louis, Missouri, USA

**Keywords:** Gq/11, uveal melanoma, proteomics, phosphoproteomics

## Abstract

Metastatic uveal melanoma (UM) patients typically survive only 2 to 3 years because effective therapy does not yet exist. Here, to facilitate the discovery of therapeutic targets in UM, we have identified protein kinase signaling mechanisms elicited by the drivers in 90% of UM tumors: mutant constitutively active G protein α-subunits encoded by GNAQ (Gq) or GNA11 (G11). We used the highly specific Gq/11 inhibitor FR900359 (FR) to elucidate signaling networks that drive proliferation, metabolic reprogramming, and dedifferentiation of UM cells. We determined the effects of FR on the proteome and phosphoproteome of UM cells as indicated by bioinformatic analyses with CausalPath and site-specific gene set enrichment analysis. We found that inhibition of oncogenic Gq/11 caused deactivation of PKC, Erk, and the cyclin-dependent kinases CDK1 and CDK2 that drive proliferation. Inhibition of oncogenic Gq/11 in UM cells with low metastatic risk relieved inhibitory phosphorylation of polycomb-repressive complex subunits that regulate melanocytic redifferentiation. Site-specific gene set enrichment analysis, unsupervised analysis, and functional studies indicated that mTORC1 and 6-phosphofructo-2-kinase/fructose-2,6-biphosphatase 2 drive metabolic reprogramming in UM cells. Together, these results identified protein kinase signaling networks driven by oncogenic Gq/11 that regulate critical aspects of UM cell biology and provide targets for therapeutic investigation.

## Highlights

• Uveal melanomas are driven by oncogenic G proteins.
• G protein q/11-specific inhibitor prevents uveal melanoma growth.
• MS phosphoproteomics analysis reveals downstream pathways.
• Oncogenic Gq/11 drives proliferation, metabolism, and dedifferentiation.

## Graphical Abstract

**Figure:**
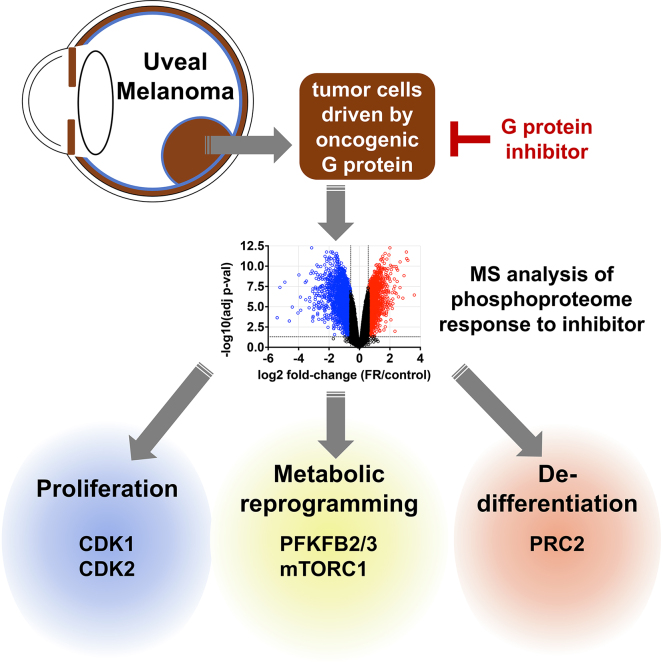

## In Brief

Uveal melanomas are highly aggressive eye tumors that are driven by mutant, constitutively active G protein alpha-q/11. We use the Gq/11-specific inhibitor FR900359 and perform MS phosphoproteomics to identify downstream targets of Gq/11 oncogenesis. We identify specific pathways and targets of phosphorylation that drive proliferation, metabolism, and dedifferentiation in Gq/11-driven uveal melanoma cells.

Uveal melanoma (UM) is the most common intraocular tumor in adults. Nearly half of UM patients develop metastatic disease, usually involving the liver, regardless of whether primary ocular tumors are treated (1). Once metastatic disease is detected, median survival is only 2 to 3 years (2) because effective therapy has yet to be developed. Therapeutic approaches including tumor resection, liver-directed therapy, or immune checkpoint therapy improve quality of life but extend survival only a few months and are not curative (3, 4). Likewise, tebentafusp, a bispecific protein consisting of an affinity-enhanced T-cell receptor fused to an anti-CD3 effector that can target T cells to UM cells, has shown positive clinical effect but without significantly improving overall survival (5).

Oncogenic signaling mechanisms in UM are being discovered and investigated clinically as therapeutic targets. Oncogenesis in 90% of UM patients initiates in melanocytes of the choroid, ciliary bodies, or iris (collectively, the uvea) upon acquisition of mutations that constitutively activate the G protein α-subunits Gαq (GNAQ) (6) or Gα11 (GNA11) (7, 8). In the remaining 10% of UM patients, uveal melanocytes acquire mutations that constitutively activate a Gq/11-coupled leukotriene receptor (CYSLTR2) (9) or a Gq/11 effector, phospholipase Cβ4 (10). Each of these oncogenic drivers in UM cells activates signal transduction networks involving PKC isoforms and Erk. Whereas PKC and Erk pathway (MEK) inhibitors arrest proliferation of UM cells (11), they have not provided significant therapeutic benefit in clinical trials (12, 13).

Although once considered undruggable, oncogenic Gq/11 has emerged recently as therapeutic targets in UM (14, 15). The highly selective Gq/11 inhibitors FR900359 (FR) and YM-254890 (YM) target UM cells driven by oncogenic Gq/11 but not those driven by oncogenic BRAF. FR arrests Gq/11-driven UM cells in the G1 phase of the cell cycle (16, 17, 18, 19), elicits melanocytic redifferentiation of UM cells with low (class 1) but not high (class 2) metastatic potential (19), inhibits growth of xenografted UM tumors (17, 19), attenuates metabolic reprogramming (20), and inhibits cytoskeletal dynamics (21) and mechanosensing important for tumor cell migration (22, 23). However, FR does not efficiently kill UM tumor cells from patients or regress UM xenografts (19). Therefore, additional targets in UM cells need to be identified and characterized.

To pursue this goal, here we have used quantitative proteomic/phosphoproteomic and bioinformatic analyses of UM cells to identify signaling mechanisms and biological processes impacted by inhibition of oncogenic Gq/11 with FR. These objectives are distinct from those of previous proteomic investigations, which analyzed the secretomes of UM tumors (24) and cell lines (25) and compared metastatic and nonmetastatic UM tumors (26, 27). Our studies highlight the use of CausalPath, a recently developed bioinformatics tool that derives causal relationships between signaling mechanisms and biological processes they control by quantitatively comparing experimental multi-omics data with curated information (28, 29).

## Experimental Procedures

### Cell Lines and Reagents

FR900359 was purified from *Ardisia crenata* according to published methods (30). The structure of purified FR900359 relative to a commercially available equivalent (UBO-QIC; University of Bonn (Germany)) was established by NMR. Torin1 (Cat# S2827) and KAN0438757 (Cat# S0400) were purchased from Selleck Chemicals. The human UM PDX-derived cell lines MP41 (ATCC Cat# CRL-3297, RRID:CVCL_4D12) and MP46 (ATCC Cat# CRL-3298, RRID:CVCL_4D13) (31) were purchased from ATCC. The human UM cell line OCM-1A (RRID:CVCL_6934) was derived by the generous gift of Dr June Kan-Mitchell (Biological Sciences, University of Texas at El Paso) (32). All cell lines were grown at 37 °C in 5% CO_2_ in RPMI 1640 medium (Life Technologies) supplemented with antibiotics and 25% fetal bovine serum (MP41 and MP46) or 10% fetal bovine serum (OCM-1A). Cells were not grown above passage 35. For proteomics analyses, cells were grown in 60-mm dishes with complete media. To harvest cells, 60-mm dishes were placed on ice, media was aspirated, and ice-cold TBS was added immediately. Cells were washed twice more with ice cold TBS to remove all traces of serum proteins and media. Cells were then scraped in fresh TBS and transferred to 15 ml conical tubes to pellet by centrifugation. TBS supernatants were removed, and the pellets were flash frozen immediately on dry ice.

### Preparation of Labeled Peptides and Phosphopeptides

Deep-scale proteomics and phosphoproteomics was performed using the previously described protocol (33) with minor modifications (34) and as described below. The cell pellets were solubilized in 200 μl of lysis buffer (50 mM Tris HCl, pH 8.0), containing 8 M urea, 75 mM NaCl, 1 mM EDTA, 10 mM NaF, phosphatase inhibitor cocktail 2 (1:100) and cocktail 3 (1:100), 2 μg/ml aprotinin, 10 μg/ml leupeptin and 1 mM PMSF, pH 8.0. Samples were transferred, using lysis buffer rinse (50 μl), to a Covaris MilliTUBE (Covaris, Cat. No. 520071) with AFA fiber for focused ultrasonication. Lysates were sonicated for 12 min (Peak Incident Power: 70 W, Duty Factor: 50%, cycles/burst: 200, time: 12 min, temp: 5–8 ˚C), placed on ice, and transferred to 1.7 ml tubes (Axygen, Cat. No. NCT-175-C). Lysates were spun at 16,000*g* in an Eppendorf centrifuge for 30 min at 4 °C. Supernatants were removed and protein concentration determined using a Pierce BCA Protein Assay Kit (Pierce, Cat. No. 23225) and protein aliquoted (350 μg) into 0.5 ml tubes and stored at −80 °C. The reference pool peptides for the cell line studies were generated by combining a lysate aliquot from all 24 samples in the study. An additional pool of all eight sample lysates from each cell line was prepared followed by tryptic digestion as described below. As previously described (33), peptides were prepared after sequential digestion with endoprotease Lys-C (Wako Chemicals, Cat. No. 129-02541) (1 mAU per 50 μg total protein) and trypsin (1:50 (wt/wt)). The peptides were purified using solid phase extraction with a SepPak (Waters, Cat. No. WAT036820). The peptides were eluted with 1.5 ml of 50% (vol/vol) MeCN (J.T. Baker, Cat. no. 9829-03) containing 0.1% (vol/vol) formic acid (Fluka, Cat. No. 94318-250 ml).

An aliquot (1%) was removed for quantification using the Pierce Quantitative Fluorometric Peptide Assay kit (Pierce, Cat. No. 23290). The remainder of the samples and reference pool sample were transferred into 1.7 ml Eppendorf tubes, lyophilized, and stored at −80 °C. The lyophilized peptides were dissolved in 40 μl of Hepes buffer (100 mM, pH 8.5) and labeled according to the vendor protocol using the tandem mass tag (TMT)-10 (Thermo Fisher Scientific, Cat. No. 90406) and TMT-11 (Thermo Fisher Scientific, Cat. No. 34808) reagent kits. The efficiency of labeling was >99% by LC-MS.

The labeled samples were quenched and combined into three plexes (see supplemental Data File S3). The TMT labeled samples were desalted using a SepPak (Waters, Cat.No. WAT036820). The eluents were collected into the 1.5 ml tubes, frozen, and lyophilized. Each of the TMT-pooled peptide plexes was separated using basic reverse-phase chromatography as previously described (33). The 96 fractions from each sample were concatenated into 24 fractions plus fraction A as previously described (33). An aliquot (5%) of each concatenated fraction and fraction A was analyzed using LC-MS. The remaining of the 24 fractions were combined to 12 fractions as previously described (33). The enrichment of phosphopeptides was performed using immobilized metal affinity chromatography and the previously described reproducible protocol (33). After enrichment, the phosphopeptides were dried in a speedvac and stored at −80 °C in the autosampler vials.

### Mass Spectrometry Data Acquisition

For LC-MS analysis, phosphopeptides were gently mixed in 9 μl of water containing 3% (vol/vol) MeCN, 0.1% (vol/vol) FA for 30 min at room temperature. Only enough samples for 24 h of data acquisition were prepared.

### Ultra-high Performance Mass Spectrometry

The samples were analyzed using ultra-high performance mass spectrometry (35) using a hybrid quadrupole Orbitrap LC-MS System, Q-Exactive PLUS interfaced to an EASY-*nano*-LC 1000. A 75 μm i.d. × 50 cm Acclaim PepMap 100 C18 RSLC column (Thermo Fisher Scientific) was equilibrated with 100% solvent A (1% FA) on the nano-LC for a total of 11 μl at 700 bar pressure. Samples in FA (1% (vol/vol)) were loaded at a constant pressure of 700 bar. Peptide chromatography was initiated with mobile phase A (1% FA) containing 5% solvent B (100% MeCN, 1% FA) for 5 min, then increased to 23% B over 100 min, to 35% B over 20 min, to 95% B over 1 min and held at 95% B for 39 min, with a flow rate of 250 nl/min. Data were acquired in data-dependent mode. Full-scan mass spectra for phosphopeptide-enriched fractions were acquired with the Orbitrap mass analyzer using a scan range of *m/z* = 350 to 1800 and a mass resolving power set to 70,000. Twelve data-dependent high-energy collisional dissociations were performed with a mass resolving power at 35,000, a fixed lower value of *m/z* 110, an isolation width of 0.7 Da, and a normalized collision energy setting of 32. The maximum injection time was 60 ms for MS1 analysis and 105 ms for MS2 analysis. Ions that were selected for MS2 were dynamically excluded for 20 s. The automatic gain control was set at a target value of 1e6 ions for MS1 scans and 1e5 ions for MS2. Full-scan mass spectra for global fractions were acquired with the Orbitrap mass analyzer using a scan range of *m/z* = 350 to 1500 and a mass resolving power set to 70,000. Twelve data-dependent high-energy collisional dissociations were performed with a mass resolving power at 35,000, a fixed lower value of *m/z* 100, an isolation width of 1.2 Da, and a normalized collision energy setting of 32. The maximum injection time was 60 ms for MS1 analysis and 120 ms for MS2 analysis. Ions that were selected for MS2 were dynamically excluded for 40 s. The automatic gain control was set at a target value of 3e6 ions for MS1 scans and 1e5 ions for MS2.

### Protein and Phosphopeptide Identification and Quantification

The machine data from the LC-MS analysis of isobaric-labeled peptides, using the Q-Exactive PLUS mass spectrometer, were converted to peak lists using Proteome Discoverer (version 2.1.0.81, Thermo Fisher Scientific). Raw MS data files were searched with MaxQuant (version 2.0.3.0) with the human SwissProt proteome containing isoforms (42,384 entries) from UniProtKB (downloaded March 2022). Whole-cell lysate and phospho-enriched fractions were searched together with the following parameters: specific trypsin digestion with up to two missed cleavages, fixed modification of carbamidomethylation, variable modifications of methionine oxidation, protein N-terminal acetylation, and STY phosphorylation (phospho-enriched fractions only) and TMT10 quantification with lot-specific correction factors. A 20 ppm precursor mass tolerance was used for the first search, followed by 4.5 ppm during the main search, and a 20 ppm tolerance was used for fragment matching. Peptide and protein identifications were controlled to a false discovery rate (FDR) of 1%. Each cell line was searched separately. MS proteomics data have been deposited to the ProteomeXchange Consortium *via* the MassIVE partner repository with the dataset identifier PXD038115. Annotated spectra for MP41, MP46, and OCM-1A experiments are available on MS-Viewer (36) with keys a7hoxcqd3v, mbvegrzjti, and rvuap5eqpi, respectively.

### Quality Assessment

The initial processing, quality assurance, and analysis of isobaric-labeled peptide LC-MS data were performed with proteoQ (version 1.5.0.0, https://github.com/qzhang503/proteoQ) software developed with the tidyverse approach ((37) tidyverse: Easily Install and Load the 'Tidyverse'; R package version 1.3.1. https://CRAN.R-project.org/package=tidyverse) with open source software for statistical computing and graphics, R (R Core Team (2021). R: A language and environment for statistical computing; (R Foundation for Statistical Computing, Vienna, Austria URL https://www.R-project.org/) and RStudio (RStudio Team (2016); RStudio: Integrated Development for R. RStudio, Inc., Boston, MA URL http://www.rstudio.com/). For initial quality assessment, the reporter-ion intensities from each plex of TMT, *m/z* values (channels) were converted to logarithmic ratios (base 2), relative to the average reporter-ion intensity of reference samples within each plex (supplemental Fig. S7). Within each sample, Dixon’s outlier removals were carried out recursively for peptides with greater than two identifying peptide-spectrum matches. The median of the ratios of peptide-spectrum matches that could be assigned to the same peptide was first taken to represent the ratios of the incumbent peptide. The median of the ratios of peptides was then taken to represent the ratios of the inferred protein. To align protein ratios across samples, likelihood functions were first estimated for the log-ratios of proteins using finite mixture modeling, assuming two-component Gaussian mixtures (38). The ratio distributions were then aligned so that the maximum likelihood of log-ratios was centered at zero for each sample. Scaling normalization was performed to standardize the log-ratios of proteins across all samples. To reduce the influence of outliers from either log-ratios or reporter-ion intensities, the values between the 5th and 95th percentile of log-ratios and 5th and 95th percentile of intensity were used in the calculations of SDs. Metric multidimensional scaling of protein log2-ratios was performed with the base R function stats:cmdscale and stats:prcomp, respectively.

### Experimental Design and Statistical Rationale

For the proteomics experiments, three cell lines were studied under two conditions with four replicates each resulting in 24 samples. A common reference pool was created from an equal mixture of all samples. Each cell line was put into its own TMT10 plex consisting of eight samples plus two reference pool channels. Downstream analysis was performed using MSstatsTMT (version 2.2.7) (39) and MSstatsTMTPTM (version 1.1.2). First, data was preprocessed using MSstatsHelper (https://github.com/GoldfarbLab/MsstatsHelper) to map phosphosites to protein group identifiers. Next, protein-level and phosphosite-level summarization of TMT quantification was performed with MSstatsTMT using global and reference channel normalization. Missing values were imputed by the MBimpute method in MSstats. Protein and phosphosite differential expression analysis was performed with the linear mixed-effects model from MSstatsTMT and MSstatsTMTPTM, using a moderated t-statistic, Benjamini-Hochberg multiple test correction, and normalization to protein abundance for phosphosites. Volcano plots were generated with the resulting adjusted *p*-values and estimated fold-changes. Fold-change thresholds were set to ±50% (log_2_FC = 0.58) due to expected ratio compression from MS2-level TMT quantification. Phosphosites were filtered out if they had <0.75% localization probability or lacked protein-level quantification. Gene set enrichment analysis was then performed using MSigDB annotations (40), while PTMSigDB annotations were used for phosphosites (41, 42, 43).

### CausalPath Analysis

We used CausalPath (29) to understand the potential cause-effect relations in the differential phosphopeptide measurements. The method uses detailed pathway knowledge as prior information and identifies chains of evidence that can causally link pairs of significant phosphopeptide changes. The method considers if a phosphorylation site is activatory or inhibitory, considers the sign of the known relation (phosphorylation *versus* dephosphorylation), makes sure the target site in prior information matches the detected site in the experiment, and evaluates if the directions of changes are aligned with the prior information using a logical equation. The result is a set of relations that collectively form a network. We applied this method as described in a recent protocol (28). For the detection of significant phosphopeptide changes, we used multiple FDR cutoffs from 0.1 to 0.0001, which resulted in networks with different sizes and confidence levels. After the network generation step, CausalPath tests the significance of protein activities on the network through label randomization followed by the assessment of the size of targets for each protein. Significant findings from this analysis are then marked on the result networks. For this test, we used a 0.1 FDR threshold. The inference of differentially active kinases in Figure 2 is the result of this test. Please note that the different FDR values specified in Figure 2 panels refer to the previous test—detection of significant phosphopeptide changes, which needs to be done prior to differential activity detection. To better interpret CausalPath results and for complexity management, we also generated subgraphs of the result networks that focused on the neighborhood of selected genes of interest.

The full CausalPath causative networks linked to this manuscript are available on Figshare.com. They can be interactively visualized through the CausalPath web server following the steps below.

(1) Extract the archive on local storage.
(2) In the Chrome Browser, go to http://causalpath.cs.umb.edu and click on “View results from a previous analysis”.
(3) Select the root folder of the extracted archive and press “Upload”. Confirm upload.
(4) The list of networks is displayed on the left. Double-click one of them to visualize.

### Immunoblots

For standard immunoblots, cells were lysed and cleared in radioimmunoprecipitation assay buffer (150 mM sodium chloride, 1% Triton-X100, 0.5% sodium deoxycholate, 0.1% sodium dodecyl sulfate, 50 mM Tris, pH 8.0) with 1× complete protease inhibitor (Roche, cat.11697498001). Lysates were resolved on 12% SDS-PAGE gels and transferred to immobilon(P) polyvinylidene difluoride membrane (Millipore, cat.IPVH00010). Membranes were blocked with 5% w/v milk in TBST (25 mM Tris pH 7.2, NaCl 150 mM, 2.7 mM KCl, 0.1% v/v Tween 20) and incubated with primary antibodies. Membranes were washed with TBST at least three times and incubated with IRDye 680 Goat anti-rabbit and IRDye 800 Goat anti-mouse (LI-COR). Following incubation, membranes were washed at least three times with TBST and signals were detected using LI-COR Odyssey model 9120 imaging system (LI-COR). Primary antibodies used for immunoblots were as follows: phospho-S6 ribosomal protein (Ser240/244) (D68F8) (Cell Signaling Technology, catalog# 5364SS lot#8) and anti-ribosomal protein S6 antibody (C-8) (Santa Cruz Biotechnology catalog# sc-74459 lot# K2520).

### Seahorse Experiments

Cells were plated on Seahorse Xfe96 cell culture microplates (Agilent Technologies) coated with Cell-Tak (Corning Life Sciences) in 50 μl of appropriate medium for each cell line or sample. Cell density was set near 80% confluency. Cells were plated 2 days prior to assay and treated with FR, torin1, or vehicle (0.1% DMSO) 18 h prior to assay. Each treatment group had a minimum of three replicate wells, and each plate had a minimum of four background wells. Seahorse XF DMEM pH 7.4 media was used to run all experiments, with a final well volume of 180 μl. Basal respiration was measured using the Mito Stress Test assay kit and glycolysis was measured using the Glycolytic Stress Test assay kit (GST), both from Agilent. Samples were run according to Agilent assay protocol on Seahorse XF96 and XFe96 analyzers. Normalization of all data to cell number/well was accomplished through measurement of DAPI fluorescence. After the Seahorse assay was completed, cells were fixed with cold methanol and stained with DAPI. Plates were read at 358/20 nm excitation and 461/20 nm emission on a Cytation 5 imaging reader using Gen5 (version 3.08) software (https://www.agilent.com/en/support/biotek-software-releases) from BioTek. Rates for glycolysis and basal respiration were gathered from Glycolytic Stress Test assay kit and Mito Stress Test assay kit report generators created by Wave Desktop Software (version 2.6.1) (https://www.agilent.com/en/product/cell-analysis/real-time-cell-metabolic-analysis/xf-software), available from Agilent, and utilized in Microsoft Excel 2016. Statistical analyses were performed in GraphPad Prism (version 8.2.1 (441)) (https://www.graphpad.com). Summary data values on all graphs represent means. Error bars on graphs represent SEM for all Seahorse experiments. Stars indicate significance as determined by statistical analysis (∗ < 0.01). Data for treated and untreated samples were analyzed *via* unpaired t-tests with Mann-Whitney post hoc tests.

## Results and Discussion

We used two PDX-derived UM cell lines, MP41 and MP46, to compare the effects of FR on UM cells that (1) recapitulate properties of UM cells from patient tumors (19, 20) that have low *versus* high metastatic potential due, respectively, to the presence (MP41) or absence (MP46) of the BAP1 metastasis suppressor (31, 44); (2) are driven by constitutively active Gα11 (GNA11-Q209L; MP41) *versus* Gαq (GNAQ-Q209L; MP46) (31); and (3) do (MP41, BAP1-positive) *versus* do not (MP46, BAP1-negative) redifferentiate into melanocytic-like cells upon inhibition of oncogenic Gq/11 (19) (Table 1). Biallelic loss of BAP1 occurs in about 47% of UM tumors from patients (1), and the metastatic potential of loss of BAP1 has been established previously using survival data from UM patients (1). We used a BRAF(V600E)-driven UM cell line (OCM-1A) as an FR-insensitive control (16, 19, 20).

**Table 1:** Characteristics of cell lines used in this study

Cell line	MP41^31^	MP46^31^	OCM-1A^32^
Source	PDX of primary eye tumor	PDX of primary eye tumor	primary eye tumor
Oncogene mutation	GNA11(Q209L)	GNAQ(Q209L)	BRAF(V600E)
BAP1 status	wt	null	wt
Metastatic potential	low	high	**-**
FR responses:			
Growth arrest^16^	**+**	**+**	**-**
Reduced metabolism^20^	**+**	**+**	**-**
Redifferentiation^19^	**+**	**-**	**-**

Reference table comparing the MP41, MP46, and OCM-1A cell lines. References for this information are cited as superscripts. Metastatic potential is based on clinical data from Gq/11-mutant UM tumor-only.

**Fig. 1:**
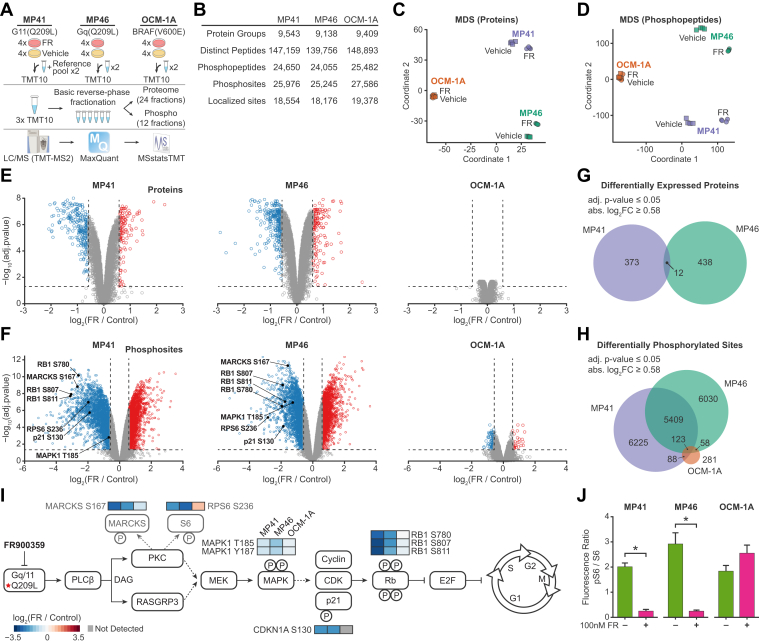
**Sample comparisons show FR responses in Gq/11-driven UM cells.***A*, two PDX-derived Gq/11-driven UM cell lines, MP41(GNA11-Q209L) and MP46(GNAQ-Q209L), and an FR-insensitive UM cell line (OCM-1A) driven by BRAF(V600E) were treated with FR (four samples each) or vehicle (four samples each) for 24 h. Proteins were isolated, digested, and TMT-labeled prior to reverse-phase fractionation. Samples were analyzed by LC/MS1 and TMT-MS2 using the Q-Exactive mass spectrometer. The raw MS data files were searched with MaxQuant, and protein and phosphosite differential expression analyses were performed with the linear mixed-effects model from MSstatsTMT and MSstatsTMTPTM, using a moderated t-statistic, Benjamini-Hochberg multiple test correction, and normalization to protein abundance for phosphosites. *B*, protein and phosphosite yields are given for each cell line. Protein and phosphoprotein log_2_-ratios were aligned across samples so that the maximum likelihood of log-ratios was centered at zero for each sample and then normalized across all samples for metric multidimensional scaling (MDS) of proteins (*C*) or phosphopeptides (*D*) to assess differences among indicated groups of samples. Volcano plots were generated with the resulting adjusted *p*-values and estimated fold changes. *E*, volcano plots of protein changes in response to FR treatment were generated separately for each cell line. *Blue* indicates significant decreases (*p* < 0.05; log_2_ fold-change < −0.58; *i.e.* fold-change <50%) and *red* indicates significant increases (*p* < 0.05; log_2_ fold-change >0.58; *i.e.* fold-change >50%) in response to FR. *F*, volcano plots of changes in relative abundance of phosphosites in response to FR were generated for each cell line. Specific phosphosites relevant to Gq/11 signaling in MP41 and MP46 cell lines (*I*) are indicated. *G*, proteins were filtered by adjusted *p*-value <0.05 and absolute log_2_(fold-change) > 0.58 and compared among cell lines. Overlaps indicate significant changes among the indicated lines in the same direction in response to FR. *H*, phosphosites were filtered as indicated and compared among cell lines. Overlaps indicate significant changes in the same direction among the indicated lines. *I*, schematic diagram indicating the flow of signaling downstream of constitutively active Gq/11. *Dotted lines* indicate indirect interactions. Changes in phosphorylation of sites indicated in (*F*) are color-coded by Log_2_(fold-change) in response to FR. Constitutively active Gq/11 (Q209L) drives phospholipase Cβ (PLCβ) to produce diacylglycerol (DAG), which, in UM cells, activates both PKC and a Ras guanine nucleotide exchange factor (RASGRP3) that activate MAPK/Erk Kinase (MEK) to phosphorylate MAPK. Active MAPK drives cell proliferation through cyclin-dependent kinases (CDKs), which phosphorylate and inactivate the Rb family of cell cycle checkpoint inhibitors. *J*, immunoblots (supplemental Fig. S8) were performed to measure independently the phosphorylation of S6 (RPS6) on serine-246 (indicated in F and I) in response to FR in each cell line and showed significantly reduced (∗ *p* < 0.01) phosphorylation in MP41 and MP46 cells. UM, uveal melanoma.

To define molecular events driven by oncogenic Gq/11 signaling, we treated UM cell lines (four independent experiments per cell line per condition) with vehicle or FR (100 nM) for time (24 h) (Fig. 1*A*). We chose this dose because it is well above the IC50 of FR to block constitutively active Gq/11 signaling in HEK293 cell reporter assays (between 1.9 and 3.8 nM) (19) and the IC50 to initiate the mechanisms culminating in G1 arrest (between 0.5 nM and 40 nM) (16, 17, 18). One hundred nanomolars of FR is also sufficient to inhibit metabolic reprogramming (20) and initiate melanocytic redifferentiation of BAP1^+^ UM cells (16, 19). Total cell lysates prepared from each sample were analyzed individually by LC-MS/MS as described in Experimental Procedures to identify the effects of inhibiting oncogenic Gq/11 on the proteome and phosphoproteome of UM cells (Fig. 1*A*).

### Global Effects of Inhibiting Oncogenic Gq/11 in UM Cells

Results indicated that FR had large effects on the phosphoproteomes of Gq/11-driven UM cells, modest effects on the total proteomes of Gq/11-driven UM cells, and small effects on BRAF-driven UM cells, as indicated by pairwise comparisons between all samples (supplemental Figs. S1 and S2), multidimensional scaling (Fig. 1, *C* and *D*), and plotting fold-change *versus* significance (Fig. 1, *E* and *F* and supplemental Data Files S1 and S2). The small effects of FR on BRAF-driven OCM-1A cells were not due to reduced protein or phosphoprotein yields compared to the other cell lines (Fig. 1B) but instead demonstrate the small effects from inhibition by FR of the WT Gq/11 expressed in these cells. Therefore, the large effects of FR on MP41 and MP46 cells were caused specifically by inhibition of oncogenic Gq/11. In support of this conclusion, nearly half of the significant proteomic and phosphoproteomic changes elicited by FR were shared between MP41 and MP46 cells, whereas few of these changes were also shared with BRAF-driven OCM-1A cells (Fig. 1, *G* and *H*). The proteomic and phosphoproteomic effects of FR that differed between MP41 and MP46 cells may be due in part to the presence *versus* absence of BAP1 because, for example, we have shown previously that BAP1 is required for FR to evoke melanocytic redifferentiation of UM cells (19).

### Regulation of Canonical Oncogenic Gq/11 Signaling and the UM Cell Cycle by FR

Phosphoproteomic data indicated that FR attenuated canonical signaling mechanisms downstream of oncogenic Gq/11 and caused G1-phase cell cycle arrest. FR significantly reduced phosphorylation (Fig. 1, *F* and *I*) of (1) a site (S167) in MARCKS phosphorylated by PKC; (2) the activation loop (T185) of Erk2 (MAPK1); (3) a site (S236) in ribosomal protein S6 (RPS6) targeted downstream of PKC; (4) sites (S780, S807, S811) in retinoblastoma protein (RB1) phosphorylated by cyclin-dependent kinases (CDKs); and (5) a site (S130) in the CDK inhibitor p21^CIP1^ (CDKN1A) phosphorylated by Erk2 to promote G1 progression. These effects of FR were confirmed by quantitative immunoblotting for phosphorylation of ribosomal protein S6 on S236 (Fig. 1*J* and supplemental Fig. S6) and the results of experiments reported previously (18, 19).

### Protein Kinases Affected by Inhibiting Oncogenic Gq/11

To gain broader understanding of signaling networks and biological processes driven by oncogenic Gq/11 signaling in UM cells, we interrogated our proteomic and phosphoproteomic data with the recently developed bioinformatics tool CausalPath. CausalPath uses curated information and statistical criteria to probe multi-omics data and infer causal relationships between signaling mechanisms and biological processes affected by experimental perturbation (28, 29). CausalPath accounts for the direction, impact, and site specificity of each potential interaction and assembles significant relations into a network.

**Fig. 2:**
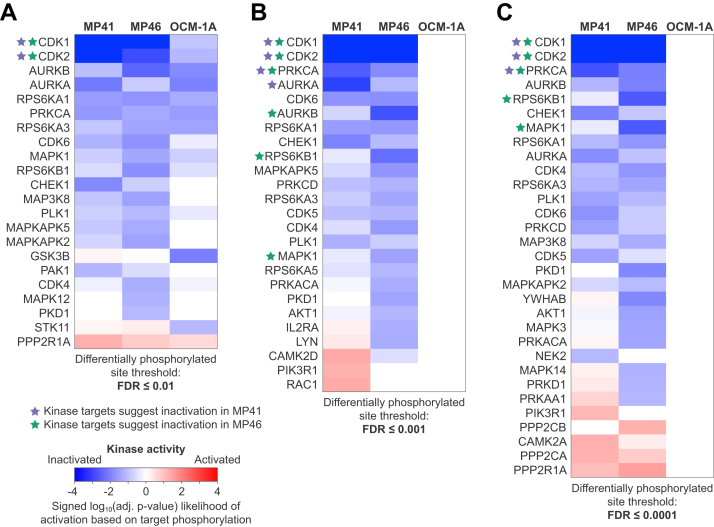
**Heatmaps of kinases regulated by FR.** Distributions of significantly differing proteins and phosphopeptides were analyzed in CausalPath to identify kinases that showed significant functional changes in response to FR. Heatmap colors correspond to the log of the adjusted *p*-value signed to indicate direction of change. *Asterisks* indicate kinases that were defined as “inactivated” by CausalPath in MP41 (*purple asterisks*) or MP46 (*green asterisks*) cells at each FDR based on significant collective reduced phosphorylation of known targets. *A*, CausalPath calculated the statistical likelihood of inactivation of kinases based on the overall significance of changes in their targets. At phosphopeptide change detection of FDR <0.01, CausalPath identified several kinases as inactivated based on response to FR in all three cell lines, including significant calls in the OCM-1A cells. *B*, increasing the stringency further to FDR < 0.001 still identified most of the same kinases as significantly inactivated in both MP41 and MP46 cell lines, whereas no kinases showed significant responses to FR in OCM-1A cells. *C*, further increasing the stringency to FDR < 0.0001 identified fewer significant kinases in MP41 but several more kinases in MP46, including CDK1, CDK2, PRKCA (PKCα), RPS6KB1 (p70-S6K1), and MAPK1 (Erk2). CDK, cyclin-dependent kinase; FDR, false discovery rate.

To determine which effects of FR were due to inhibition of oncogenic *versus* wild type Gq/11, we used CausalPath at several statistical thresholds (FDR). At low stringency (FDR < 0.01), CausalPath detected significant effects of FR in UM cells driven by oncogenic forms of either Gq/11 or BRAF (Fig. 2*A*). The small effects of FR on BRAF-driven UM cells likely were due to inhibition of WT Gq/11 because they were not observed at higher stringency (FDR < 0.001 or <0.0001) (Fig. 2, *B* and *C*). In contrast, CausalPath analysis even at high stringency identified significant effects of FR in UM cell lines driven by oncogenic Gq/11, as indicated by inactivation of CDK1, CDK2, and protein kinase Cα (PKCα; PRKCA) signaling networks (Fig. 2*B*). Interestingly, CausalPath identified a higher number of significant results at more stringent thresholds (Fig. 2). This results from using two independent statistical tests: the first one to identify significant changes—what is up and what is down—at the given thresholds; the second one to identify differentially active kinases based on those up/down phosphopeptides. The latter test uses a fixed stringency (FDR < 0.1), which generates more significant results when the up/down inferences have higher precision.

CausalPath also suggested that FR had somewhat different effects in the two Gq/11-driven UM cell lines. For example, FR caused inactivation of Aurora kinase A (AURKA) in MP41 cells and Aurora kinase B (AURKB) in MP46 cells (Fig. 2*B* and supplemental Figs. S3 and S4), while UM cells are sensitive to inhibitors of both Aurora kinases A and B (45). Differences such as these suggested that analyzing phosphoproteomic data to determine whether FR changed activity of specific signaling networks and their linked biological process could be challenging due to complexity of the data, statistical factors, or regulation of substrate phosphorylation by multiple protein kinases and phosphatases. These considerations prompted us to evaluate the performance of CausalPath as described below.

### Evaluating CausalPath as a Tool to Detect Signaling Network Regulation

**Fig. 3:**
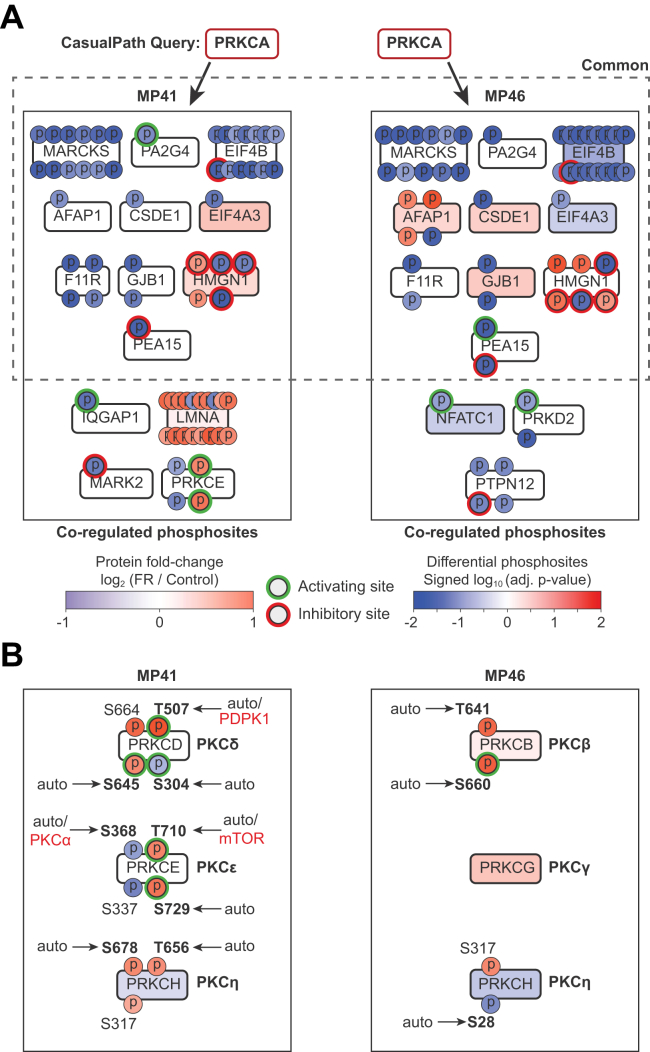
**CausalPath analysis of PKC regulation by FR in UM cells.***A*, CausalPath generated subgraphs for PKCα (PRKCA) in both MP41 and MP46 UM cell lines, based on loss of phosphorylation of known targets in response to FR at FDR ≤ 0.001. Phosphorylation sites are *red* if increased with FR and *blue* if decreased. Color intensities for phosphorylation are based on adjusted *p*-values, as determined by CausalPath. Sites outlined in *green* are activation by phosphorylation, and those outlined in *red* are inhibited by phosphorylation. Protein levels are color coded based on log2 fold change with FR (*red*: increased; *blue*: decreased). Kinases outlined in *red* were inactivated by FR. *Black* boxes indicate coregulated targets of phosphorylation as defined by CausalPath for each cell line. *Dashed* box indicates targets of phosphorylation that are regulated in common between both cell lines. *B*, manual interrogation of other PKC isoforms identified by CausalPath in each cell line. Phosphosites in bold are known targets of autophosphorylation; other kinases that are known to target specific sites are indicated in *red*. Activities of these isoforms are determined by DAG and Ca^2+^ levels, such that these phosphosites are not specifically indicative of either activation or inactivation. FDR, false discovery rate; UM, uveal melanoma.

We evaluated CausalPath performance by examining the detailed effects of FR on phosphorylated proteins linked to the canonical downstream effectors PKCα (PRKCA) and Erk2 (MAPK1) in Gq/11-driven MP41 and MP46 cells. CausalPath indicated that FR reduced phosphorylation of established PKC substrates MARCKS, EBP1 (PA2G4), and EIF4B (46) (Fig. 3*A*). CausalPath was able to indicate that PKCα was inactivated even though FR had complex effects on substrates phosphorylated by PKCα and other protein kinases, as indicated in MP46 cells where FR decreased AFAP1 phosphorylation on a PKCα site (S277) (Fig. 3*A* and supplemental Data File S1) (47) and increased phosphorylation of AFAP1 on sites unlinked to PKCα (Fig. 3*A* and supplemental Data File S1).

However, CausalPath did not indicate that FR caused inactivation of novel PKC isoforms PKCδ (PRKCD) and PKCε (PRKCE) known to be driven by oncogenic Gq/11 signaling in UM cells (48). Inactivation of PKCδ and PKCε by FR might be difficult to distinguish from PKCα inactivation because diagnostic substrates sufficient to distinguish between PKCα and novel PKCs have yet to be identified or are less well represented in curated phosphoproteomic datasets. Nevertheless, CausalPath was able to suggest that PKCδ and PKCε were functionally competent (Fig. 3*B*) because FR increased rather than decreased phosphorylation at C-terminal sites that prime these novel PKC isoforms for activation by DAG (49).

**Fig. 4:**
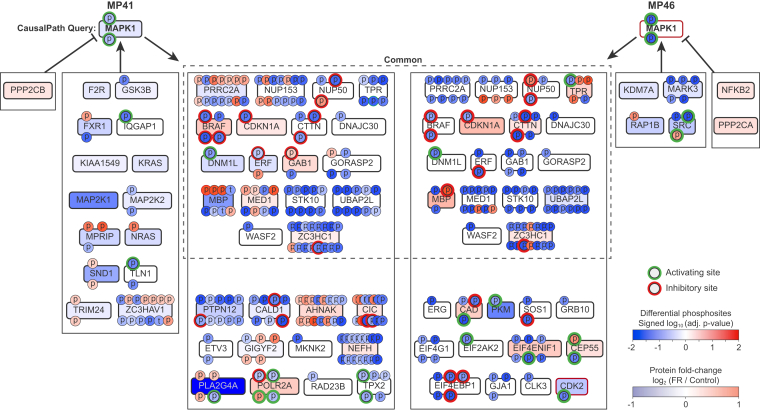
**CausalPath analysis of Erk2 targets in response to FR.** CausalPath subgraphs for Erk2 kinase (MAPK1) responses to FR in both MP41 and MP46 cell lines at FDR ≤ 0.001. Phosphorylation sites are *red* if increased with FR and *blue* if decreased. Color intensities for phosphorylation are based on adjusted *p*-values, as determined by CausalPath. Sites outlined in *green* are activation by phosphorylation, and those outlined in *red* are inhibited by phosphorylation. Protein levels are color coded based on log2 fold change with FR (*red*: increased; *blue*: decreased). Kinases outlined in *red* were inactivated by FR. *Black* boxes indicate coregulated targets of phosphorylation as defined by CausalPath for each cell line. *Dashed* box indicates targets of phosphorylation that are regulated in common between both cell lines. Several Erk targets, including PRRC2A, NUP153, MED1, GAB1, MBP, and TPR, showed differential patterns of overall phosphorylation between the two cell lines. Most of these differentially phosphorylated sites were linked to other kinases, whereas sites known to be associated with Erk2 showed significant dephosphorylation in both lines. FDR, false discovery rate; MBP, myelin basic protein.

To analyze Erk regulation by FR, we used CausalPath in two ways. When used conventionally at each statistical threshold (FDR), CausalPath indicated that FR inactivated Erk2 (MAPK1) in MP46 cells but not in MP41 cells (Figs. 2 and 4) because the *p*-value CausalPath calculated for the inactivation of Erk2 in the MP41 cells was 0.05 (when differential abundance FDR <0.01), which was above the significance threshold when multiple hypothesis testing was considered. By comparison, prior evidence shows that FR inactivates Erk in both cell lines (Fig. 1, *F* and *I* and (18, 19)). As an alternative, we used CausalPath simply to generate Erk2 signaling networks and then inspected them manually. These analyses suggested that FR did indeed inactivate Erk2 in MP41 cells, as indicated by reduced phosphorylation of established Erk sites in PRRC2A (S1219), NUP153 (S614), NUP50 (S221), PTPN12 (S571), CALD1 (S759 and S789), and TPR (S2155) (Fig. 4).

In contrast, CausalPath did not indicate that FR augmented protein kinase activity even though phosphorylation of many proteins was increased (Fig. 1*F*). To address this question independently of CausalPath, we examined 27 representative proteins whose phosphorylation at identified sites was increased most significantly by FR (supplemental Tables S1 and S2). Phosphorylation of myelin basic protein on T232, an *in vitro* Erk1/2 site (50, 51), was the only FR-augmented site in these 27 proteins that had been linked to a specific protein kinase (supplemental Tables S1 and S2) as indicated by searches of PhosphositePlus and iPTMnet. Thus, additional approaches would be required to determine how inhibition of oncogenic Gq/11 increases phosphorylation of certain proteins to regulate UM cell function.

### CDK Targets Affected by Inhibiting Oncogenic Gq/11 in UM Cells

CausalPath indicated that CDK1 and CDK2 were strongly inactivated by FR in UM cells (Fig. 2). Because CDK1 and CDK2 phosphorylate checkpoint inhibitors to progress, respectively, through G2-M and G1-S transitions in the cell cycle, CDK2 inactivation is expected to be a proximal effect of inhibiting oncogenic Gq/11 that arrests UM cells in G1, whereas CDK1 inactivation would be a distal consequence of FR-induced G1 arrest.

**Fig. 5:**
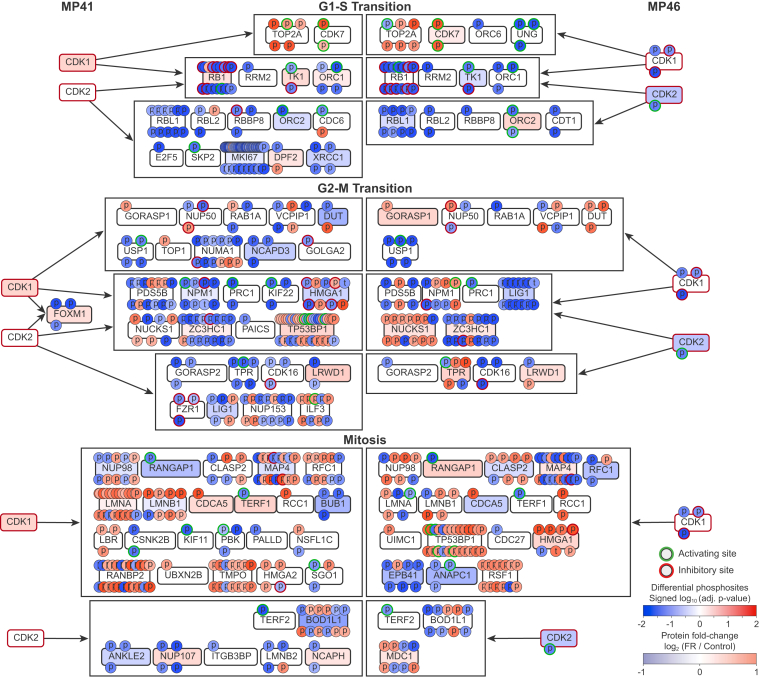
**Combined CausalPath subgraphs for CDK1 and CDK2.** Combined subgraphs for CDK1 and CDK2 were generated in CausalPath for both MP41 and MP46 cell lines in response to FR at FDR ≤ 0.001. Color intensities for phosphorylation are based on adjusted *p*-values, as determined by CausalPath. Sites outlined in *green* are activation by phosphorylation, and those outlined in *red* are inhibited by phosphorylation. Protein levels are color coded based on log2 fold change with FR (*red*: increased; *blue*: decreased). Kinases outlined in *red* were inactivated by FR. *Black* boxes indicate coregulated targets of phosphorylation by CDK1, CDK2, or both kinases as defined by CausalPath for each cell line. Targets of phosphorylation defined by CausalPath were entered into the MSigDB analysis portal and sorted by Pathway. Unsorted targets were then resorted by Hallmark and then by GO:BP terms. CausalPath identified CDK targets with regulatory and catalytic functions in G1-S checkpoint progression and G2-M checkpoint progression and mitosis. CausalPath also identified significant dephosphorylation of many noncell cycle targets of CDKs involved in mitogenic signaling, chromatin remodeling, and regulation of the actin cytoskeleton (supplemental Fig. S5). CDK, cyclin-dependent kinase; FDR, false discovery rate.

To suggest how CDK inactivation by FR affects UM cell function, we analyzed networks assembled by CausalPath at high stringency (FDR ≤ 0.001) (Fig. 5). These networks contained many targets of CDK1 and/or CDK2, including RB1, TK1, and NPM1, that are critical for progression through G1-S, G2-M, or mitosis and showed significantly reduced phosphorylation in FR-treated MP41 and MP46 UM cells (Fig. 5). Several targets involved in cell cycle regulation were assigned to both CDK1 and CDK2, whereas others were linked only to CDK1 or CDK2 (Fig. 5). These networks should be interpreted cautiously because certain CDK-target assignments are biologically inappropriate (*e.g.* G1/S targets assigned to CDK1), likely due to certain limitations of curated data used by CausalPath. With this point in mind, we examined CausalPath-generated networks for evidence of non-cell cycle proteins targeted by FR-induced CDK1/2 inactivation. This analysis identified targets involved in PI3K/AKT signaling, chromatin remodeling, and actin cytoskeleton regulation (supplemental Fig. S5), which suggest new mechanisms that could be investigated by future studies.

### Signaling Mechanisms Correlated Positively or Inversely with the Effects of FR on Gq/11-Driven UM Cells

**Fig. 6:**
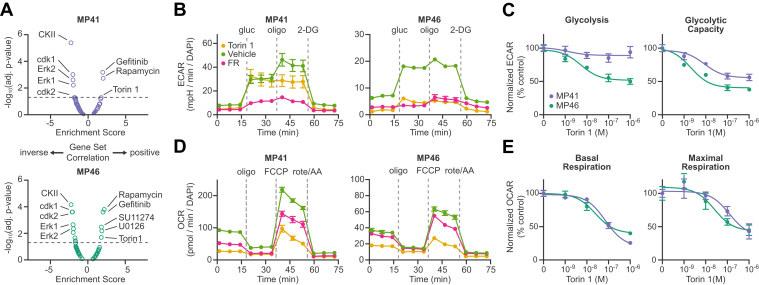
**Potential metabolic regulators downstream of FR.***A*, site-specific gene set enrichment analysis (ssGSEA) was performed to interrogate the PTM signature databases (PTMsigDB) for both kinases and perturbations and plotted as nominal enrichment score (ES) *versus* -Log(adjusted *p*-value) for each cell line. CDK and Erk kinase activities were inversely correlated with FR treatment. The mTOR inhibitors rapamycin and torin1 were positively correlated with FR treatment. This analysis also identified a significant correlation with the EGFR inhibitor gefitinib, the MEK inhibitor, U0126, and the cMet inhibitor, SU11274 in MP46 cells. *B*, Torin-1 is an ATP-competitive mTORC1/mTORC2 dual inhibitor. Responses of MP41 and MP46 cells to torin1 were compared to FR in Agilent Seahorse assays for glycolytic stress. *C*, dosage responses to torin1 were measured in glycolysis and glycolytic capacity. MP41 cells showed no significant change in glycolysis but significant change in spare capacity (glycolytic capacity – glycolysis). *D*, responses of MP41 and MP46 cells to torin1 were likewise compared to FR in Agilent Seahorse assays for mitochondrial respiration stress. *E*, dosage responses to torin1 were measured in basal and maximal respiration and demonstrated highly similar responses in both cell lines. CDK, cyclin-dependent kinase; PTM, posttranslational modification.

As an independent means of analyzing our phosphoproteomic data, we performed site-specific gene set enrichment analysis. Here, we used the posttranslational modification signature database (PTMsigDB) to determine whether the effects of FR on the phosphoproteomes of Gq/11-driven UM cells correlated positively or inversely with curated signatures of drug perturbations, kinase activities, and signaling pathways. Results (Fig. 6*A*) indicated that the effects of FR in Gq/11-driven UM cells correlated (1) inversely with CDK and Erk activity, as expected from CausalPath analysis (Figs. 4 and 5) and prior evidence (16, 17, 18); (2) positively with effects of an MEK inhibitor (U0126), as expected (16, 17, 18); (3) inversely with casein kinase 2 (CKII; CSNK2A1) activity, which had not been suggested by CausalPath or prior evidence; (4) inversely with the curated effects of the mTOR inhibitors rapamycin and torin1; and (5) inversely with curated effects of indirect inhibitors of the PI3K–Akt–mTOR pathway (gefitinib, an EGFR inhibitor (52, 53); and SU11274, a cMet inhibitor (54)). Because mTOR signaling is a key regulator of metabolic activity, this pathway may be required for oncogenic Gq/11 to drive metabolic reprogramming in UM cells (20, 55), which we tested below.

### Metabolic Reprogramming Driven by Gq/11 in UM Cells Involves mTOR

We determined whether mTORC1 activity is required for oncogenic Gq/11 signaling to drive metabolic reprogramming in UM cells. These experiments were based on our prior studies (20) showing that FR strongly attenuates glycolytic and respiratory activity in UM cells driven by oncogenic Gq/11 but has no effect on BRAF-driven UM cells. Accordingly, we compared the effects of FR and torin 1, which inhibits kinase-dependent functions of mTORC1 and mTORC2 without causing feedback activation of PI3K/AKT signaling as can occur with rapamycin (56), in glycolytic (Fig. 6, *B* and *C*) and mitochondrial (Fig. 6, *D* and *E*) stress tests with Gq/11-driven UM cells.

Results indicated that FR and torin 1 had similar but somewhat distinct effects on metabolic activity (Fig. 6, *B*–*E*). In MP46 cells, FR and torin 1 inhibited glucose-stimulated glycolysis and glycolytic capacity (Fig. 6, *B* and *D*, and (20)). In MP41 cells, torin 1 had insignificant effect on glucose-stimulated glycolysis but strongly inhibited glycolytic capacity (Fig. 6, *B* and *D*), whereas FR inhibited both processes in these cells (20). In contrast, basal and maximal respiration in both UM cell lines were inhibited more strongly by torin1 than FR (Fig. 6, *B* and *D*). Because the effects of FR and torin 1 were similar but distinct in certain respects, oncogenic Gq/11 and mTOR signaling may cooperate in nonlinear networks to drive metabolic reprogramming in UM cells.

### Metabolic Reprograming in Gq/11-Driven UM Cells Requires PFKFB2

We searched for additional mechanisms of oncogenic Gq/11-driven metabolic reprogramming by examining the effects of FR on UM cell proteomes and phosphoproteomes. We focused on the enzyme family that controls the commitment step in glycolysis (57): 6-phosphofructo-2-kinase/fructose-2,6-bisphosphatases (PFKFBs). PFKFB-1 through PFKFB-4 are homodimeric, bifunctional enzymes that synthesize and degrade fructose-2,6-bisphosphate (F2,6BP). F2,6BP, in turn, allosterically activates 6-phosphofructo-1-kinase, the rate-limiting enzyme in glycolysis. PFKFB isoforms are known to be phosphorylated on several sites by a variety of protein kinases to regulate their kinase:phosphatase ratios, thereby controlling F2,6BP levels and glycolytic activity.

**Fig. 7:**
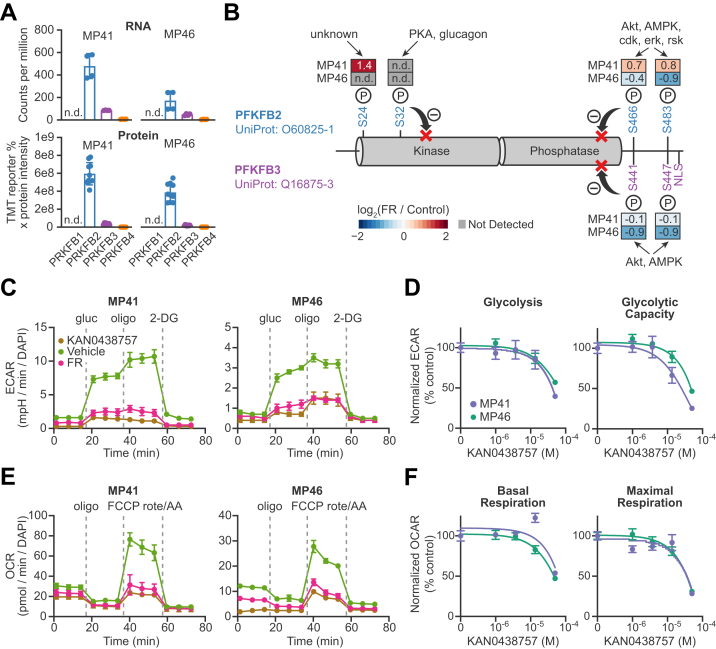
**The PFKFB inhibitor KAN0438757 regulate metabolic activity in UM cells.** The homodimeric 6-phosphofructo-2-kinase/fructose-2,6-bisphosphatases (PFKFBs) are bifunctional enzymes that synthesize and degrade fructose-2,6-bisphosphate in glycolysis. PFKFB2 is the major cytosolic isoform of PFKFB in UM cells. *A*, expression of PFKFB isoforms in MP41 and MP46 cells based on previous RNAseq analyses (19) and this proteomics analysis. PFKFB1 expression is not detectable (n.d.) in UM cells. PFKFB2 shows the highest expression followed by PFKFB3 and then PFKFB4 has the lowest expression. *B*, schematic diagram of PFKFBs showing the locations of phosphorylation sites in PFKFB2 (*above*) and PFKFB3 (*below*). Boxes show log_2_(fold-change) in phosphorylation of each site in response to FR in the indicated cell line. S32 phosphorylation was not detectable in either cell line, and S24 phosphorylation was not detectable in MP46 cells. *C*, KAN0438757 is a PFKFB inhibitor with inhibitory activities against PFKFB3 (IC_50_ 0.2 μM) and PFKFB2 (IC_50_ ∼ 50 μM). Responses of MP41 and MP46 cells to 50 μM KAN0438757 were compared to FR in Agilent Seahorse assays for glycolytic stress. *D*, dosage responses to KAN0438757 were measured in glycolysis and glycolytic capacity. Both cell lines showed significant changes in glycolysis and glycolytic capacity only at 50 μM KAN0438757. *E*, responses of MP41 and MP46 cells to 50 μM KAN0438757 were likewise compared to FR in Agilent Seahorse assays for mitochondrial respiration stress. *F*, dosage responses to KAN0438757 were measured in basal and maximal respiration and demonstrated highly similar responses in both cell lines. UM, uveal melanoma.

We studied the effects of FR on PFKFB2 and PFKFB3 because PFKFB4 was poorly expressed and PFKFB1 was undetectable in MP41 and MP46 cells (Fig. 7*A*). The PFKFB3 splice variants expressed in UM cells ((Uniprot: Q16875-3 and Q16875-1) (19)) contain a C-terminal nuclear localization signal (58), potentially suggesting a compartment-specific function for this enzyme.

Results indicated that FR affected PFKFB2 and PFKFB3 differently in MP41 *versus* MP46 cells. In MP41 cells, FR reduced PFKFB2 protein expression and increased phosphorylation at a site (S24) of unknown function but near the key phosphorylation site (S32) that inhibits kinase activity (59) and increased PFKFB2 phosphorylation on sites (S466 and S483) that inhibit phosphatase activity and facilitate 14-3-3β (YWHAB) binding (60, 61) (Fig. 7*B* and supplemental Data File S1). The net impact of these effects on PFKFB2 activity was difficult to predict given the unknown function of S24 phosphorylation. In contrast, PFKFB3 protein expression and phosphorylation in MP41 cells were unaffected by FR.

In MP46 cells, oncogenic Gq/11 signaling targeted both PFKFB2 and PFKFB3. FR reduced phosphorylation of inhibitory sites in the phosphatase-regulatory domains of both enzymes (S466 and S483 in PFKFB2; S441 and S447 in PFKFB3) (Fig. 7*B* and supplemental Data File S1), suggesting that inhibition of oncogenic Gq/11 decreased the kinase:phosphatase ratio of both enzymes, which is predicted to reduce F2,6BP levels and attenuate glycolysis.

To determine whether PFKFB2 and/or PFKFB3 are required for metabolic reprogramming in Gq/11-driven UM cells, we compared the effects of FR and KAN0438757 (KAN), an inhibitor of PFKFB kinase activity (57), in glycolytic and mitochondrial stress tests. The respective functions of PFKFB2 and PFKFB3 were determined by using KAN at low levels to target PFKFB3 (IC_50_ 0.2 μM (57)) *versus* at high level to inhibit all isoforms including PFKFB2 (IC_50_ ∼50 μM (57)). Results obtained with MP41 and MP46 cells indicated that KAN at low level (<10 μM) had little or undetectable effect on glycolytic (Fig. 7*D*) and respiratory activity (Fig. 7*F*). By contrast, KAN at high level (50 μM) had large effects, quantitatively similar to FR in MP41 and MP46 cells on glucose-driven glycolysis and glycolytic capacity (Fig. 7, *C* and *D*) and basal and maximal respiration (Fig. 7, *E* and *F*). These results indicated that metabolic reprogramming driven by oncogenic Gq/11 in UM cells with either low or high metastatic potential requires the kinase activity of PFKFB2. They also raised the possibility that, in UM cells with high metastatic potential, oncogenic Gq/11 signaling may drive PFKFB3 activity to support metabolism in the nucleus (58). Sensors that detect metabolic activity in the nucleus would be required to address this hypothesis.

### Oncogenic Gq/11 Signaling Regulates Phosphorylation of Polycomb-Repressive Complexes that Control UM Cell Differentiation

Lastly, we investigated how oncogenic Gq/11 signaling impacts mechanisms that regulate UM cell differentiation by interrogating our proteomic and phosphoproteomic data. We focused on subunits of polycomb-repressive complexes 1 and 2 (PRC1 and PRC2), which regulate UM cell differentiation and metastatic potential. Our objective was to investigate how Gq/11 signaling antagonizes PRC2-mediated melanocytic differentiation in class 1 UM tumor cells (*e.g.* MP41(19)), which have low metastatic potential, but not in class 2 UM tumor cells (*e.g.* MP46 (19)), which have high metastatic potential due to loss of BAP1, a deubiquitinase that opposes histone H2A ubiquitination by PRC1. Accordingly, we hypothesized that inhibition of oncogenic Gq/11 with FR would have distinct effects on PRC1 and PRC2 complexes in MP41 *versus* MP46 cells.

**Fig. 8:**
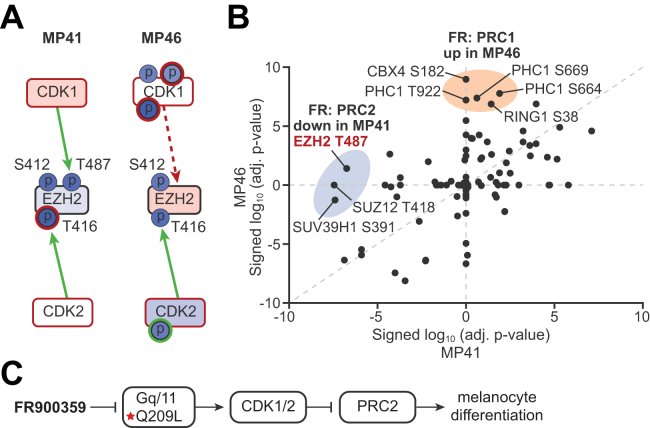
**Differential phosphorylation of polycomb group proteins in class 1 *versus* class 2 UM cells.** FR induces redifferentiation *via* PRC2 re-activation in low-risk, class1 MP41 UM cells but does not induces redifferentiation in high-risk, class 2 MP46 UM cells. *A*, direct interrogation of EZH2 in CausalPath identified two sites of significantly reduced phosphorylation (Ser412 and Thr416) in both MP41 and MP46 cells but identified significant reduction in Thr487, which CausalPath attributed to CDK1 activity, only in MP41 cells. *B*, Log(*p*-values) signed by direction of change of phosphosites in PRC1 and PRC2 polycomb group proteins were compared between MP41 and MP46 cells. FR induced loss of phosphorylation on specific sites of the PRC2 core components (highlighted in *blue*) only in MP41 cells, whereas FR induced increased phosphorylation of PRC1 components (highlighted in *orange*) only in MP46 cells. These changes suggest a shift in polycomb complex activities associated with loss of BAP1 polycomb deubiquitinase activity in MP46 cells. *C*, model of Gq/11 regulation of dedifferentiation of class 1 UM cells through CDK-dependent inactivation of PRC2 core proteins. CDK, cyclin-dependent kinase; PRC, polycomb-repressive complex; UM, uveal melanoma.

To address this hypothesis, we first used CausalPath to examine EZH2, the catalytic subunit of PRC2 complexes. Analysis of MP41 cells indicated that FR significantly decreased EZH2 phosphorylation on three sites (Fig. 8*A*): (1) T416, a CDK2 site that maintains EZH2 activity (62); (2) S412, a site of unknown function; and (3) T487, a CDK1 site that inhibits PRC2 function by interfering with core complex assembly with SUZ12 and EED (63), thereby promoting EZH2 ubiquitination, proteosome targeting, and degradation (64). In second approach, manual inspection of our phosphoproteomic data indicated that FR significantly decreased phosphorylation of the three core subunits of PRC2 complexes in class 1 MP41 cells but not class 2 MP46 cells (Fig. 8*B*). Here, FR reduced phosphorylation of the following: (1) EZH2 on T487, which, as noted above, is a CDK1 site that inhibits EZH2 activity (63, 64); (2) SUZ12 on T481, a site of unknown function; and (3) SUV39H1 on a CDK2 site (S391) that inhibits function by promoting dissociation from chromatin (65). These results suggested for the first time that class 1 UM cells such as MP41 are redifferentiated by FR because inhibition of oncogenic Gq/11 inactivates CDK1 and CDK2, thereby relieving inhibition of PRC2 complexes (Fig. 8*C*).

Polycomb complexes were targeted by FR quite differently in class 2 MP46 cells. One key distinction was that FR did not reduce phosphorylation of EZH2 on T487 (Fig. 8*A*), suggesting that PRC2 remained inhibited even though CDK1 was inactivated by FR in MP46 cells. Another striking difference observed preferentially in class 2 MP46 cells was that FR increased phosphorylation of the three core subunits of canonical PRC1 complexes, PHC1, CBX4, and RING (Fig. 8*B*). These results raised the possibility that FR-induced phosphorylation of canonical PRC1 complexes potentially reinforces rather than inhibits PRC1 function because FR does not redifferentiate class 2 UM cells (19).

## Conclusions

We undertook these studies with the objective of identifying protein kinase signaling networks regulated by oncogenic Gq/11 that potentially provide novel therapeutic targets in UM tumor cells. Our results confirmed the importance of the canonical Gq/11–PKC–Erk pathway in UM cells and identified important roles for mTOR and PFKFB2 in metabolic reprogramming and CDK regulation of PRC2 in UM cell redifferentiation. Our studies demonstrated the utility and challenges of using bioinformatics tools such as CausalPath to analyze complex proteomic and phosphoproteomic datasets. Our data and CausalPath analyses are freely available as resources for further investigation of UM.

## Data Availability

Raw MS proteomics data and MaxQuant data have been deposited to the ProteomeXchange Consortium *via* the MassIVE partner repository with the dataset identifier PXD038115. All processed data are contained within the manuscript and in the supplemental data.

## Supplemental data

This article contains 2 supplemental Tables S1 and S2, 7 supplemental Figs. S1–S7, and 3 supplemental Data Files S1–S3. Additional supplemental data are available at Figshare.com.

## Conflicts of interest

M. D. O. and K. J. B. are listed as co-inventors on a provisional patent application on TARGETED PHARMACOLOGICAL THERAPEUTICS IN UVEAL MELANOMA that is owned by Washington University in St. Louis. All other authors declare no competing interests.

## Supplementary Data

Onken_Proteomics_Figures_Suppl_04

Onken_Proteomics_Data1

Onken_Proteomics_Data2

Onken_Proteomics_expt_smry_3
