# Supplementary material for: Protein Kinase Signaling Networks Driven by Oncogenic Gq/11 in Uveal Melanoma Identified by Phosphoproteomic and Bioinformatic Analyses
Source: Mol Cell Proteomics. 2023 Sep 19;22(11):100649. doi: 10.1016/j.mcpro.2023.100649 (PMC10616553; doi:10.1016/j.mcpro.2023.100649)
Supplement: Onken_Proteomics_Figures_Suppl_04 [file mmc1.pdf]

MP41

Protein	Uniprot_ID	Site	log_2FC	adj. pvalue	Kinase
PPP6R3	Q5H9R7-2	S645	2.2	2.8E-07	unknown
AFF3	P51826	S881	2.5	5.1E-11	unknown
ADD1	P35611-3	S617	2.6	4.0E-09	unknown
SCRIB	Q14160	S37	2.6	8.3E-08	unknown
RASAL2	Q9UJF2-2	S877	2.6	3.4E-08	unknown
RHBDF2	Q6PJF5-2	S137	2.7	9.3E-09	unknown
MBP	P02686	T232	2.8	3.6E-11	ERK2
TMPO	P42166	T154	2.8	5.8E-09	unknown
RHBDF2	Q6PJF5-2	S142	3.2	4.5E-07	unknown
KCNJ13	O60928	T327	3.2	9.1E-08	unknown
VEPH1	Q14D04-2	S430	3.5	5.3E-11	unknown

Table S1: Top sites showing increased phosphorylation in MP41 cells in response to FR.

MP46

Protein	Uniprot_ID	Site	log_2FC	adj. pvalue	Kinase
LYN	P07948	S11	2.0	5.5E-13	unknown
ATP7A	Q04656-5	S362	1.9	4.2E-12	unknown
ADD1	P35611-3	S617	2.0	7.4E-09	unknown
KMT2C	Q8NEZ4-3	S759	2.3	1.1E-02	unknown
AHNAK	Q09666	T5824	2.4	2.4E-07	unknown
RHBDF2	Q6PJF5-2	S137	2.4	1.1E-10	unknown
PRPF6	O94906	T266	2.5	2.0E-10	unknown
NIPBL	Q6KC79	S709	2.5	7.7E-09	unknown
CUL3	Q13618	S737	2.5	3.1E-10	unknown
SYNM	O15061	S592	2.6	6.1E-07	unknown
LRCH1	Q9Y2L9-2	T568	2.7	4.4E-08	unknown
ATP2B4	P23634-6	S1126	2.9	8.7E-07	unknown
TOP2A	P11388	S1471	2.9	1.5E-08	unknown
KCNJ13	O60928	T327	3.1	1.8E-12	unknown
AGBL2	Q5U5Z8	Y332	3.1	1.2E-11	unknown
NAV3	Q8IVL0-3	S1186	3.2	1.9E-11	unknown
ATP2B4	P23634-6	S328	3.6	3.6E-07	unknown

Table S2: Top sites showing increased phosphorylation in MP46 cells in response to FR.

Pair-wise Correlations (proteins)

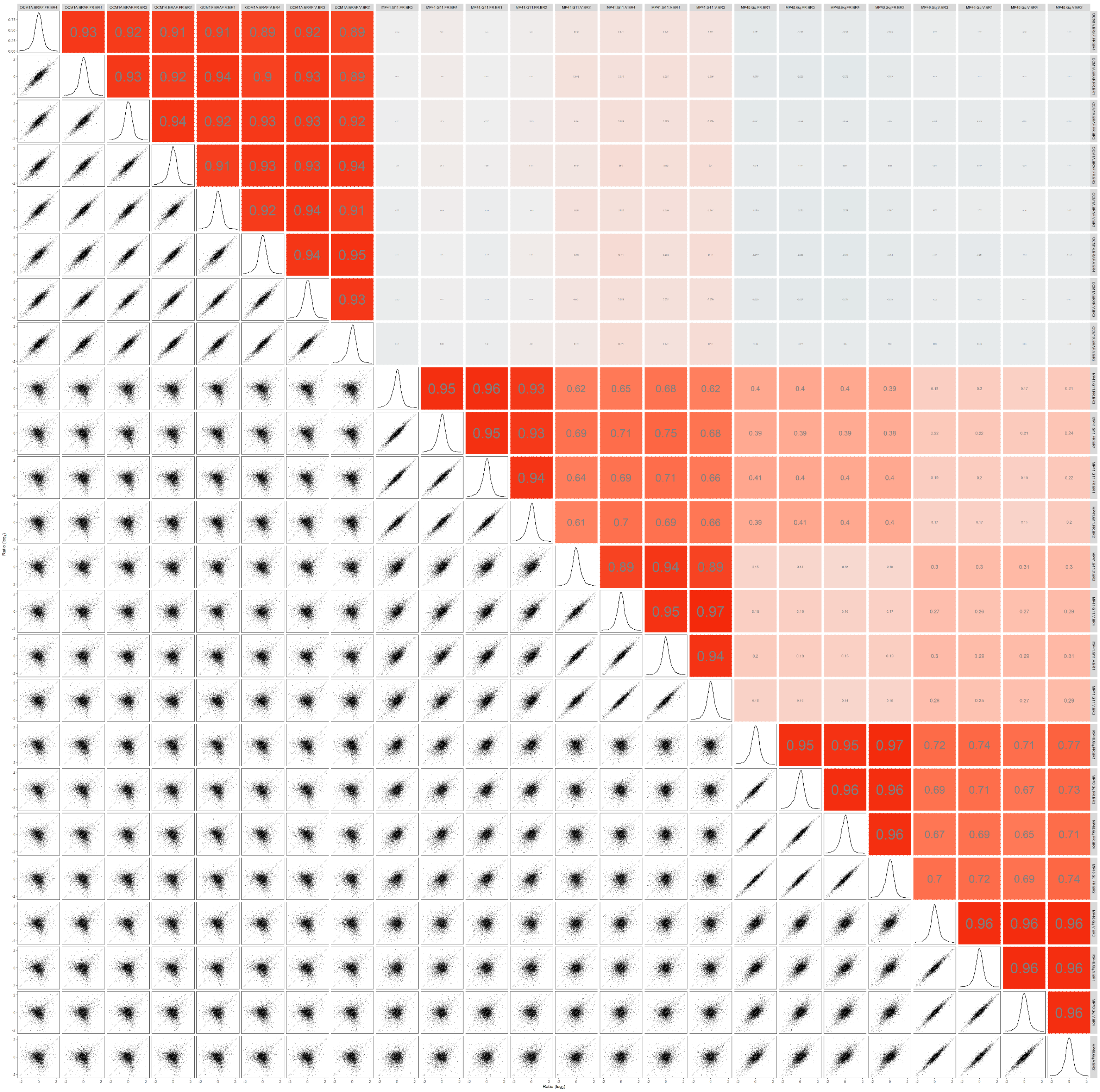

Figure S1: Sample comparisons show FR responses in Gq/11-driven UM cells. The machine data from the LC-MS analysis of isobarically-labeled peptides were converted to peak lists, and the raw MS data files were searched with MaxQuant. Protein log2-ratios were aligned across samples so that the maximum likelihood of log-ratios was centered at zero for each sample and then normalized across all samples and compared pair-wise.

Pair-wise Correlations (phosphopeptides)

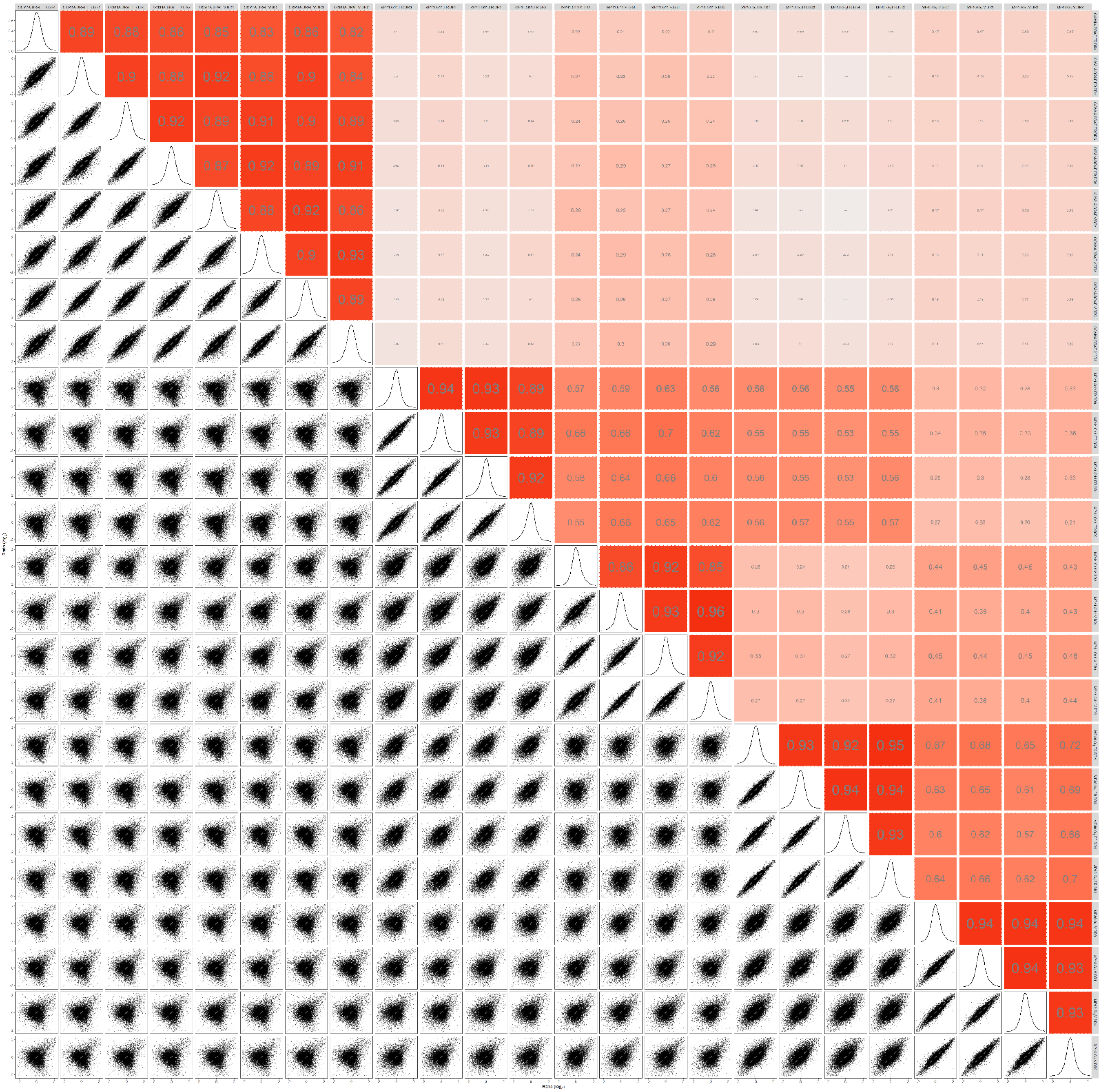

Figure S2: Sample comparisons show FR responses in Gq/11-driven UM cells. The machine data from the LC-MS analysis of isobarically-labeled peptides were converted to peak lists, and the raw MS data files were searched with MaxQuant. Phosphopeptide log2-ratios were aligned across samples so that the maximum likelihood of log-ratios was centered at zero for each sample and then normalized across all samples and compared pair-wise.

MP41 CausalPath network FDR < 0.001

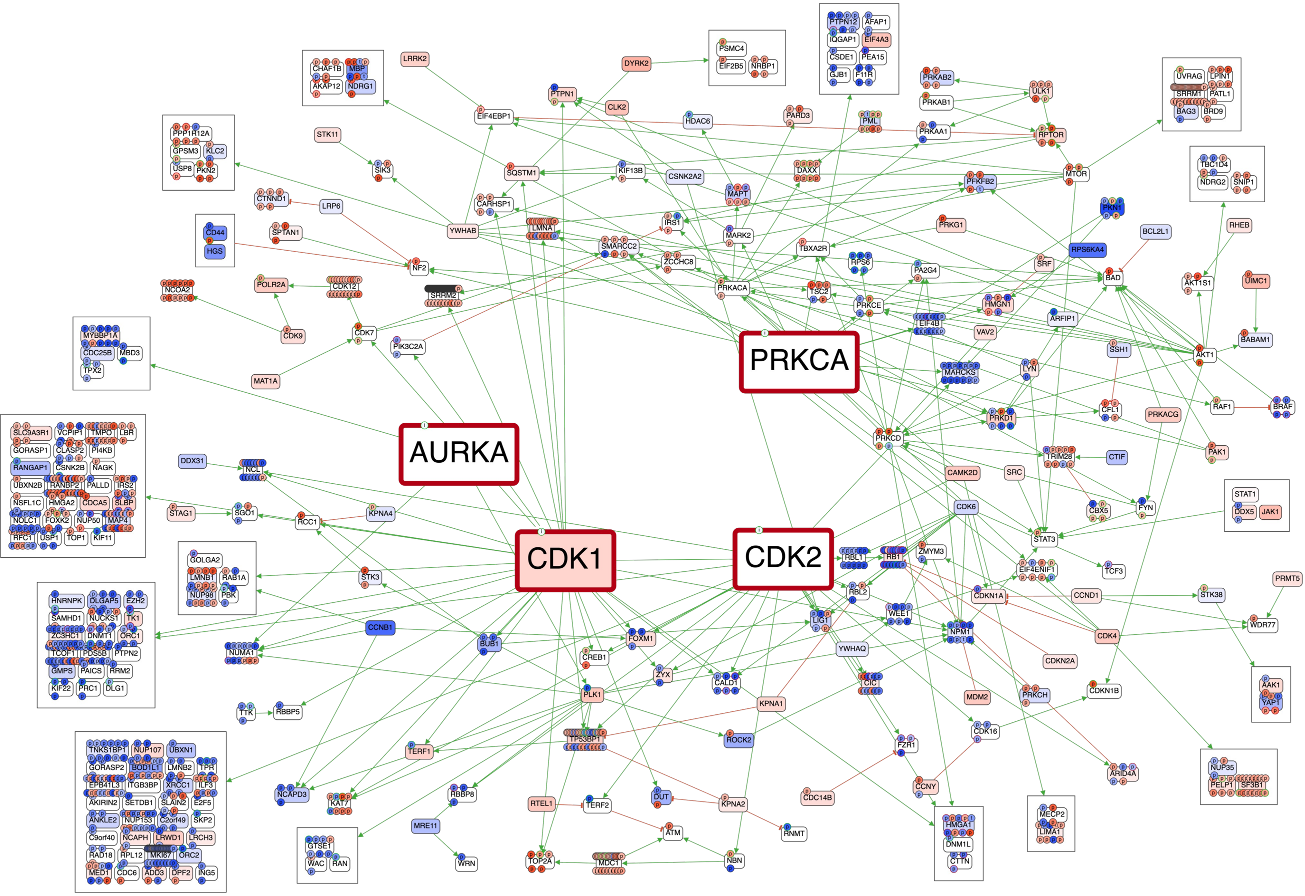

Figure S3: CausalPath analysis of MP41 cells in response to FR. CausalPath causative network for responses to FR in the MP41 cell line at FDR ≤ 0.001. Phosphorylation sites are red if increased with FR and blue if decreased. Color intensities for phosphorylation are based on adjusted p-values, as determined by CausalPath. Protein levels are color coded based on log2 fold change with FR (red: increased; blue: decreased). Black boxes indicate co-regulated targets of phosphorylation. Inactivated kinases are highlight with large boxes.

MP46 CausalPath network FDR < 0.001

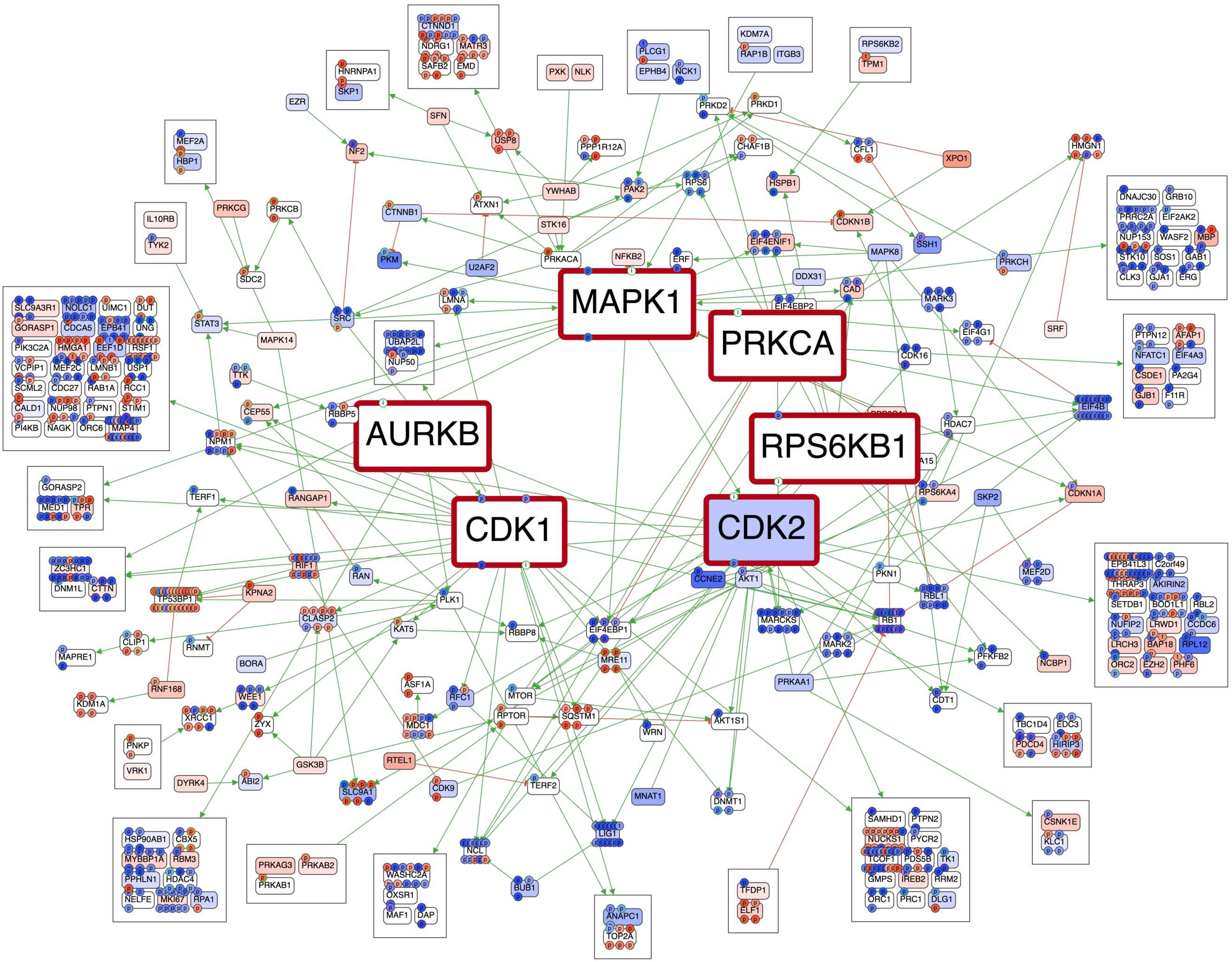

Figure S4: CausalPath analysis of MP46 cells in response to FR. CausalPath causative network for responses to FR in the MP46 cell line at FDR ≤ 0.001. Phosphorylation sites are red if increased with FR and blue if decreased. Color intensities for phosphorylation are based on adjusted p-values, as determined by CausalPath. Protein levels are color coded based on log2 fold change with FR (red: increased; blue: decreased). Black boxes indicate co-regulated targets of phosphorylation. Inactivated kinases are highlight with large boxes.

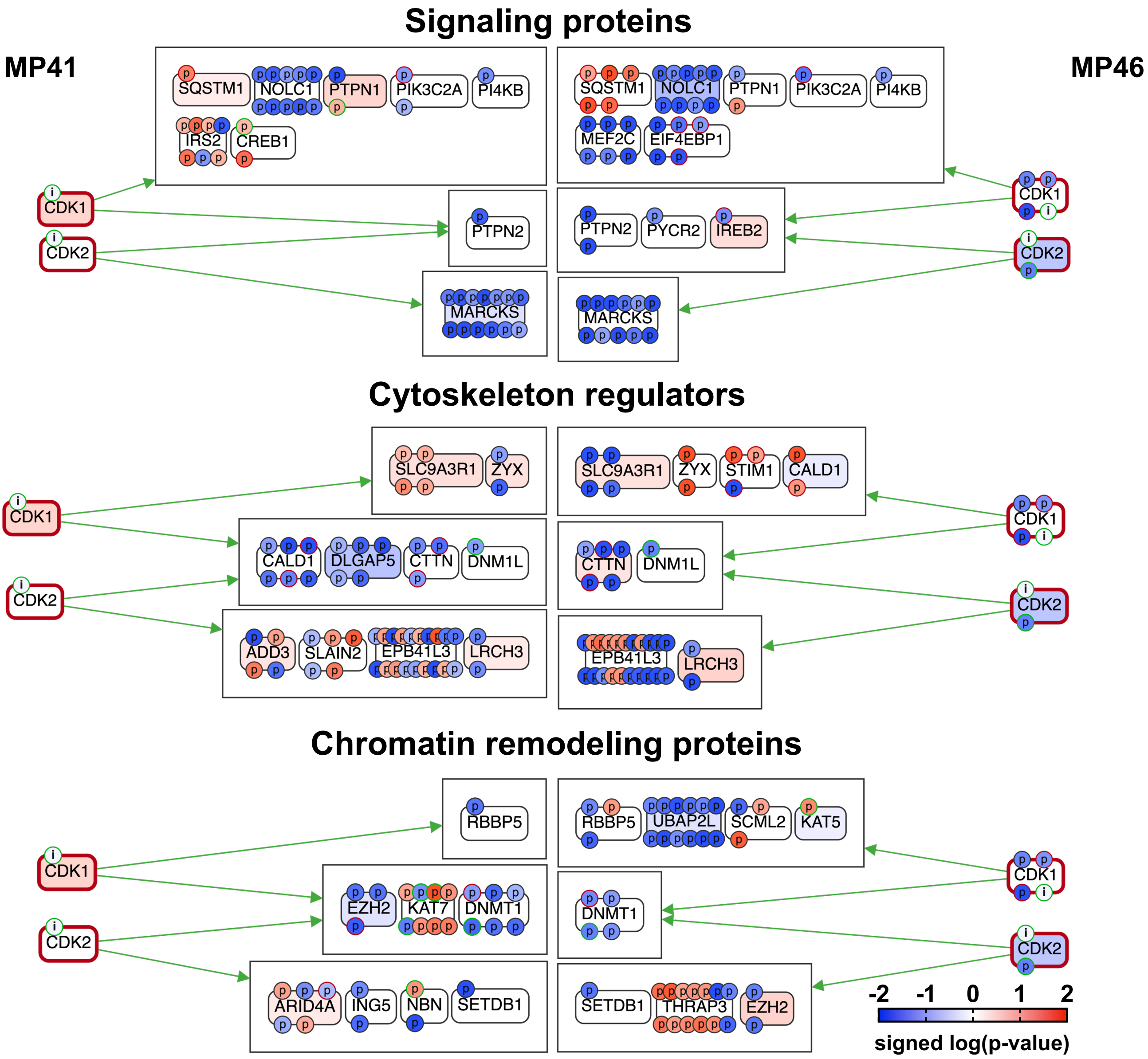

Figure S5: Combined CausalPath subgraphs for CDK1 and CDK2. Combined subgraphs for CDK1 and CDK2 were generated in CausalPath for both MP41 and MP46 cell lines in response to FR at FDR ≤ 0.001. Color intensities for phosphorylation are based on adjusted p-values, as determined by CausalPath. Protein levels are color coded based on log2 fold change with FR (red: increased; blue: decreased). Black boxes indicate co-regulated targets of phosphorylation by CDK1, CDK2, or both kinases as defined by CausalPath for each cell line. Targets of phosphorylation defined by CausalPath were entered into the MSigDB analysis portal and sorted by Pathway. Unsorted targets were then re-sorted by Hallmark and then by GO:BP terms. CausalPath identified significant dephosphorylation of many non-cell cycle targets of CDKs involved in mitogenic signaling, chromatin remodeling, and regulation of the actin cytoskeleton.

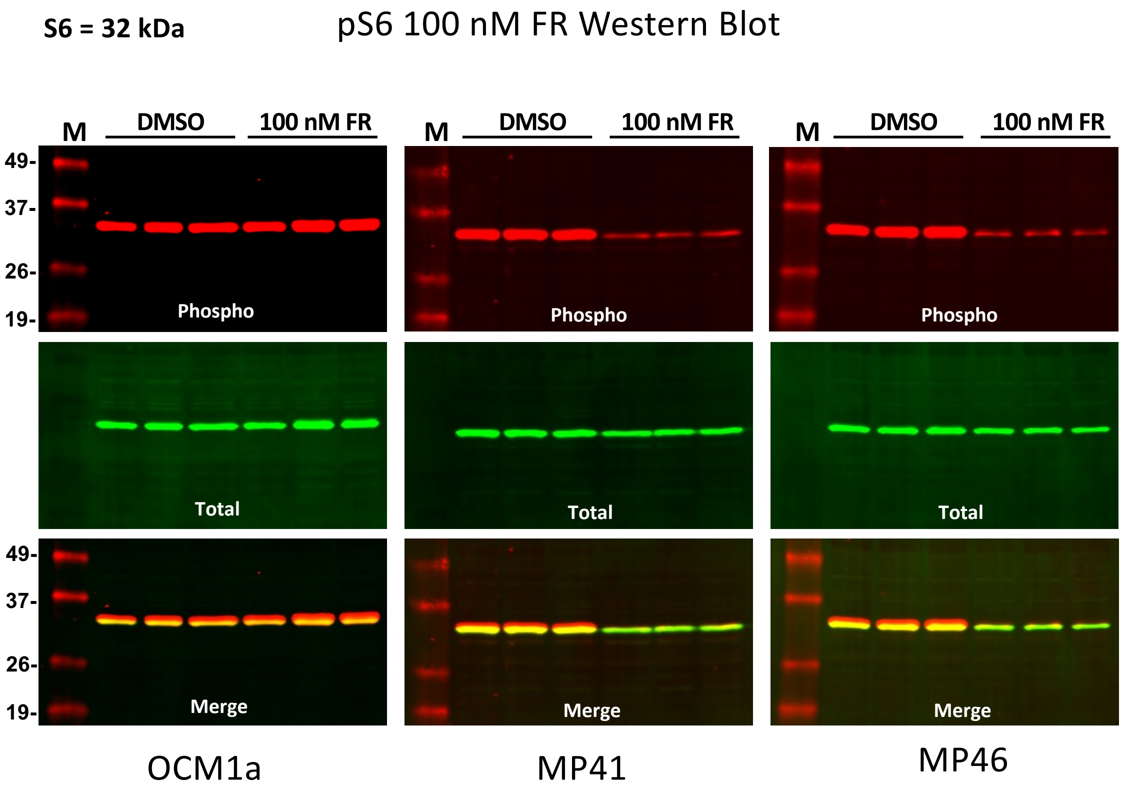

Figure S6: Fluorescence imaging scans for RPS6 immunoblots. OCM-1A, MP41, and MP46 cells were treated with 100 nM FR or vehicle (DMSO) for 24 hours in three separate experiments each (biological replicates). Cell lysates were normalized to protein content by Bradford assay and equal amounts were loaded to each of six lanes per gel. Lysates were resolved on 12% SDS-PAGE gels, transferred to PVDF membranes, and incubated with rabbit anti-phospho-S6 ribosomal protein (Ser240/244) and mouse anti-ribosomal protein S6 antibodies. Membranes were imaged using IRDye 680 Goat anti-rabbit and IRDye 800 Goat anti-mouse secondary antibodies on a LI-COR Odyssey imaging system.

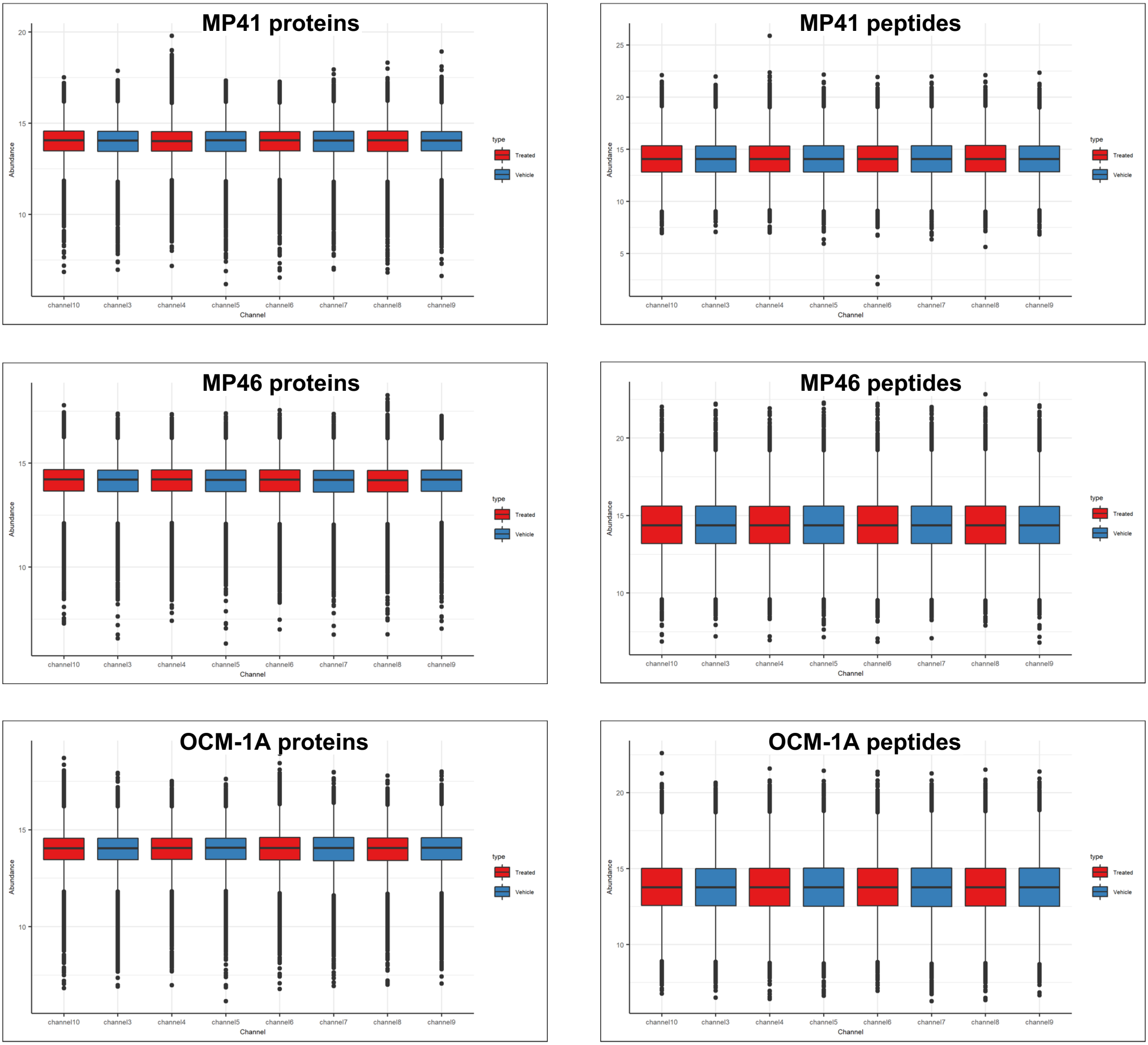

Figure S7: Boxplots of protein and phosphopeptide abundance. Summary data from MaxQuant analysis of protein and phosphopeptide abundances for MP41, MP46, and OCM-1A cell lines. Each channel represents a single sample. Boxes are bounded by quartile values for each sample (the box extends from the 25th to 75th percentiles), with the middle black bar at the median value for each sample. Control samples are indicated by blue boxes and FR-treated samples are indicated by red boxes.
